# The Effects of a Digital, Transdiagnostic, Clinically and Peer-Moderated Treatment Platform for Young People With Emerging Mental Health Complaints: Repeated Measures Within-Subjects Study

**DOI:** 10.2196/50636

**Published:** 2023-12-13

**Authors:** Marilon van Doorn, Anne Monsanto, Chen Lu Wang, Sander C J Verfaillie, Thérèse A M J van Amelsvoort, Arne Popma, Monique W M Jaspers, Ferko Öry, Mario Alvarez-Jimenez, John F Gleeson, Dorien H Nieman

**Affiliations:** 1 Amsterdam University Medical Centers (Location AMC) Amsterdam Netherlands; 2 Amsterdam Public Health Research Institute Amsterdam Netherlands; 3 Antes Rotterdam Netherlands; 4 GGZ inGeest Specialized Mental Health Care Amsterdam Netherlands; 5 Department of Psychiatry and Neuropsychology Maastricht University Maastricht Netherlands; 6 Department of Medical Informatics Amsterdam Public Health Research Institute Amsterdam University Medical Centers (Location AMC) Amsterdam Netherlands; 7 Buurtzorg Jong Almelo Netherlands; 8 Centre for Youth Mental Health The University of Melbourne Melbourne Australia; 9 Orygen Melbourne Australia; 10 Healthy Brain and Mind Research Centre and School of Behavioural and Health Sciences Australian Catholic University Melbourne Australia

**Keywords:** indicative prevention, youth mental health, Moderated Online Social Therapy, MOST+, eHealth, well-being, early detection and intervention, Engage Young People Early, ENYOY

## Abstract

**Background:**

To address the growing prevalence of youth mental health problems, early intervention is crucial to minimize individual, societal, and economic impacts. Indicative prevention aims to target emerging mental health complaints before the onset of a full-blown disorder. When intervening at this early stage, individuals are more responsive to treatment, resulting in cost-effective outcomes. The Moderated Online Social Therapy platform, which was successfully implemented and proven effective in Australia, is a digital, peer- and clinically moderated treatment platform designed for young people. The Netherlands was the first country outside Australia to implement this platform, under the name Engage Young People Early (ENYOY). It has the potential to reduce the likelihood of young people developing serious mental health disorders.

**Objective:**

This study aims to investigate the effects on young people using the ENYOY-platform in relation to psychological distress, psychosocial functioning, and positive health parameters.

**Methods:**

Dutch-speaking young people with emerging mental health complaints (N=131) participated in the ENYOY-platform for 6 months in a repeated measures within-subjects study. Psychological distress, psychosocial functioning, and positive health parameters were assessed at baseline and 3, 6, and 12 months. Repeated measures ANOVA was conducted and adjusted for age, sex, therapy, and community activity. The Reliable Change Index and Clinically Significant Index were computed to compare the baseline with the 6- and 12-month measurements. The missing data rate was 22.54% and the dropout rate 62.6% (82/131).

**Results:**

The primary analysis (77/131, 58.8%) showed that psychological distress decreased and psychosocial functioning improved over time with large effect sizes (*P*<.001 in both cases; η_p_^2^=0.239 and 0.318, respectively) independent of age (*P*=.76 for psychological distress and *P*=.48 for psychosocial functioning), sex (*P*=.24 and *P*=.88, respectively), therapy activity (*P*=.49 and *P*=.80, respectively), or community activity (*P*=.59 and *P*=.48, respectively). Similarly, secondary analyses (51/131, 38.9%) showed significant effects of time on the quality of life, well-being, and meaningfulness positive health parameters (*P*<.05; η_p_^2^=0.062, 0.140, and 0.121, respectively). Improvements in all outcome measures were found between baseline and 3 and 6 months (*P≤*.001-.01; *d*=0.23-0.62) and sustained at follow-up (*P*=.18-.97; *d*=0.01-0.16). The Reliable Change Index indicated psychological distress improvements in 38% (39/102) of cases, no change in 54.9% (56/102) of cases, and worsening in 5.9% (6/102) of cases. Regarding psychosocial functioning, the percentages were 50% (51/102), 43.1% (44/102), and 6.9% (7/102), respectively. The Clinically Significant Index demonstrated clinically significant changes in 75.5% (77/102) of cases for distress and 89.2% (91/102) for functioning.

**Conclusions:**

This trial demonstrated that the ENYOY-platform holds promise as a transdiagnostic intervention for addressing emerging mental health complaints among young people in the Netherlands and laid the groundwork for further clinical research. It would be of great relevance to expand the population on and service delivery of the platform.

**International Registered Report Identifier (IRRID):**

RR2-10.1186/s12888-021-03315-x

## Introduction

### Background

Mental health problems in young people have never been more prevalent in the Netherlands [1]. In 2021, approximately 1 in 4 young adults (aged 18-25 y) were labeled “psychologically unhealthy” (scored <60 on a screening instrument for mental health complaints) [1,2]. Worldwide, the COVID-19 pandemic has been associated with further increases in psychological complaints in the past few years [3].

Of all mental health problems, 75% start before the age of 25 years, and mental health problems are the leading contributors to disease burden among young people [4,5]. The onset of mental health problems in early adulthood poses a significant threat to subsequent personal development [6-8]. Youth mental health problems are precursors to physical health problems (eg, respiratory, cardiovascular, and infectious diseases [9]) and more severe mental health problems later in life [10]. Owing to the disruptiveness of youth mental health problems, it is estimated that they are associated with 10-fold higher societal costs during adulthood compared with mental health problems emerging in adulthood [11]. Because of the growing waitlist for mental health care [12-15] and the need for a formal classification to receive treatment in the Netherlands [16], care is often unavailable; presenting problems tend to worsen during the waiting time; and, subsequently, more intensive treatment is needed. As mental health problems are the single highest source of global economic burden (with the Netherlands estimated to have spent €4.7 billion [US $5.02 billion] in 2019 [17]), prevention and treatment of mental health complaints in youth should be the top priority to help young people fulfill their potential.

To reduce the individual, societal, and economic impacts of mental health problems among youth, it is of importance to intervene as early as possible. Indicative prevention—intervening at an early stage, namely, in people with *emerging* mental health problems and *before* the onset of a full-blown disorder—is more effective or cost-effective as, at this stage, fewer psychosocial and neurobiological consequences occur and individuals need fewer treatment sessions as they are still more responsive to treatment [18]. This approach has been adopted in many other fields of medicine, such as cardiology and oncology, and has been shown to improve prognosis [19,20]. With the purpose of adopting this approach for mental health problems, the clinical staging model was developed [21-23] (Textbox 1 [21,24]). The stages in the model differentiate emerging mental health problems with mild symptoms and functional impairment (stage 1a) or moderate symptoms and functional impairment (stage 1b) [25-27] from more discrete (stage 2), recurrent (stage 3), and treatment-resistant (stage 4) disorders. The model has been found to be usable in clinical practice and has excellent interrater reliability [21,28,29].

**Clinical staging model (adapted from Hickie et al [21]; see also the study by van Doorn et al [24]). Italics indicate the stages we focus on in this study.**

**Stage 0**

- Asymptomatic individuals at risk of a disorder who have not yet presented for care

**Stage 1a**

- *Help-seeking individuals with mild symptoms and mild functional impairment*

**Stage 1b**

- *People with attenuated syndromes with partial specificity, often with mixed or ambiguous symptoms and moderate functional impairment*

**Stage 2**

- People with discrete disorders: clear episodes of psychotic, manic, or severe depressive symptoms

**Stage 3**

- People with recurrent or persistent disorders

**Stage 4**

- People with severe, treatment-resistant, or unremitting disorders

As emerging mental health complaints (stages 1a and 1b) are often diffuse, have various trajectories, and are underpinned by overlapping mechanisms [21,24], a transdiagnostic approach could potentially add to the effectiveness of preventive interventions [30]. For example, transdiagnostic cognitive behavioral therapy (CBT) aims to pinpoint fundamental cognitive behavioral processes that are believed to be significant across various disorders and formulate a treatment strategy aimed at these shared factors [31]. Transdiagnostic treatment protocols for youth based on CBT and third-wave behavior therapies such as acceptance and commitment therapy, dialectical behavior therapy, meta-cognitive therapy, and mindfulness-based interventions are available and promote change through a common lens that applies across emotional disorders, including anxiety, depression, obsessive-compulsive disorders, and others [32,33]. Transdiagnostic models are more personalized, implementable, and scalable and, therefore, are ideal for the large-scale implementation of complex interventions [30,34]. Several meta-analyses indicate that, relative to diagnostic-specific interventions, transdiagnostic protocols are at least as or more effective at addressing the primary clinical diagnosis, more effective at alleviating comorbid diagnoses, easier to implement, and more engaging [35-41]. Transdiagnostic CBT has proven its efficacy, extending even into preventive approaches [33,42,43] and with promising preliminary results when delivered digitally [44,45]. Similarly, Păsărelu et al [46] provided evidence on the feasibility and efficacy of an internet-delivered intervention for anxiety and depressive disorders in adolescents based on rational motive behavior therapy.

Following the introduction of the clinical staging model, early intervention has been successfully implemented in Australia. There are >150 Headspace centers where young people with emerging mental health complaints can receive stepped and evidence-based care [47-49]. Moreover, usually when a young person turns 18 years, the division between child, adolescent, and adult mental health services hampers the continuity of care and often leaves them without professional help [50-52]. Headspace has successfully bridged this gap by providing services to young people between the ages of 12 and 25 years [52,53].

Given the barriers to access, the initial step toward seeking help is a difficult one. Only 30% of young people in Western countries seek help for their mental health problems [54,55], and most receive mental health care at a much later stage, when mental health symptoms have worsened or become chronic [55-58]. Barriers that contribute to the low rates of help seeking [9,59,60] include mental health stigma or self-stigma [61,62], mental health services that are not aligned with young people’s needs [50,51], lack of accessible help, the preference for solving one’s own problems, downgrading one’s problems (“others need it more than me”), and lack of knowledge about mental health (services) [55,63].

Offering digital, nonstigmatizing, and accessible care could help overcome some of these barriers. A recent scoping review found that digital indicated preventive interventions hold promising potential in (1) reducing a range of mental health problems, (2) enhancing aspects of positive health such as mental well-being and resilience, and (3) having high levels of usability and acceptability. An important observation was that web-based interventions that combined both clinical and peer moderation tended to yield the most consistent and impactful results. Nevertheless, significant gaps have been identified in the literature. For instance, there is an absence of comprehensive transdiagnostic approaches, a lack of clear definitions of emerging mental health complaints, and a lack of suitable assessment tools for emerging mental health symptoms. In addition, studies have mostly focused on either children aged <18 years or adults aged >18 years, thereby accentuating the existing gap between child and adult psychiatry. In addition, there was a scarcity of studies with follow-up data [64].

These gaps have largely been addressed in the Moderated Online Social Therapy (MOST) platform, which is a digital treatment platform moderated by both peers and clinical professionals for young people with emerging mental health issues and has been implemented in Australia. On the MOST platform, young people can (1) undertake evidence-based therapy exercises (when and where they want), (2) have one-on-one sessions with psychologists and youth with lived experience with mental health problems (peer workers), and (3) have peer-to-peer support on the platform’s community page [65,66]. On the basis of a transdiagnostic approach, MOST’s guided therapy journeys are evidence-based therapy pathways rooted in CBT, acceptance and commitment therapy, compassion-focused therapy, mindfulness-based cognitive therapy, meta-cognitive therapy, and social cognition strategies and cater to specific mechanisms underlying common complaints such as anxiety, social anxiety, social functioning, and depression. Using a process-focused approach, the intervention addresses key cognitive and behavioral processes (ie, repetitive negative thinking, cognitive affective biases, avoidance, and emotional regulation) common across mental health presentations [66]. These journeys are individually and personally tailored to individual strengths and complaints through the use of an embedded algorithm. The constructed therapy journey comprises psychoeducation and therapeutic exercises proven to be effective for specific shared mechanisms that underlie conditions such as anxiety. Furthermore, these journeys remain adaptable under the guidance of clinical moderators, ensuring a closer alignment with the unique needs of each individual [66,67]. In >13 years of research, the platform has been associated with improvements in psychological distress, perceived stress, psychological well-being, depression, (social) anxiety, loneliness, and suicidal ideation in young people with complaints across the diagnostic spectrum [66,68-70]. Moreover, there have been significant increases in vocational and educational recovery and reduced rates of hospital admissions and visits to emergency services, as well as high levels of feasibility, acceptability, engagement, and safety [71,72].

Inspired by MOST, the Netherlands was the first country outside Australia to implement the MOST platform under the name Engage Young People Early (ENYOY). ENYOY aims to support young people (aged 16-25 y) with emerging mental health complaints (Textbox 1, stages 1a and 1b) and reduce their chance of developing a serious mental health disorder [24]. Research has already shown adequate to high usability rates, and young people have reported that they considered ENYOY to be a user-friendly, safe, accessible, and inclusive initiative that helped them reduce their mental health complaints and improve their quality of life [67]. Moreover, providing users with a smartwatch to transform physiological parameters into an observable signal (“biocueing”) and offering stress-regulating exercises on the platform has been found to help them become more emotionally aware [73].

### Objectives

In this paper, we report outcomes from the ENYOY-platform using a participatory design in a within-group approach, focusing on the following parameters: psychological distress, psychosocial functioning, and positive health parameters (such as well-being and quality of life) [24]. Our hypothesis was that ENYOY would attain similar results of improvement in psychological distress [74] and psychosocial functioning [75] as have been found for the young people visiting the Headspace centers in Australia [49], as well as a positive change in positive health parameters [76]. It was expected that spending more time on the platform and being more active in the digital community would be associated with increased improvements. In addition, individual changes (using the Reliable Change Index [RCI] and Clinically Significant Index [CSI]) were calculated to gain a nuanced understanding of intervention impact at the individual level [77,78]. These indexes could help gain insights into the specific individuals who experienced significant changes and those who did not, thus enabling a more nuanced understanding of the intervention’s impact, which could contribute to a more personalized and effective treatment approach in the future. Finally, potential differences in effects between individuals in clinical stages 1a and 1b were investigated for explorative purposes. This project has the potential to realize the goal of specialized, precise, and digitally integrated treatment by providing cost-effective, nonstigmatizing, constantly available support to young people with emerging mental health complaints in the Netherlands.

## Methods

### Study Context and Design

This study took place within the context of the ENYOY project [24]. The study setting was a safe digital treatment environment, and all measurements were conducted via videocall. This study had a repeated measures within-subjects participatory design. Participants engaged with the platform for a duration of 6 months, during which measurements occurred at baseline, 3, and 6 months. Furthermore, a follow-up assessment was conducted at 12 months. The inclusion of the 3-month assessment time point was intended to explore potential dose- and time-dependent effects, namely, to ascertain whether prolonging the time on the platform would result in more substantial improvements.

### Participants

The study included Dutch-speaking young people aged 16 to 25 years with emerging mental health complaints (stages 1a and 1b; Textbox 1) who were able to provide informed consent. Those without mental health problems (stage 0), with more severe mental health problems (stage ≥2), or with an acute risk of self-harm or suicide were excluded [24]. An operationalization of the clinical staging model by Hickie et al [21] was used. Hickie et al [21] provide clear descriptions and cutoffs that were used to ascertain the clinical stage. Subsequent to the initial assessment, in which a clinical interview was conducted, the research assistant determined the clinical stage, followed by a definitive consensus reached during a weekly collaborative meeting involving a team comprising researchers, psychologists, registered mental health care and clinical psychologists, and youth with lived experience. At the time of writing this paper, an assessment tool with a digital algorithm that holds promise for use in practice had been developed and is currently under review [79].

The sample size of 43 was based on a comparable study in Australia [49] (a priori analysis with 80% power and an α of .01). Dropout rates for digital interventions for young people with emerging mental health complaints have ranged from 2% to 73% [64]. A sample size of 125 at baseline was estimated to give us ample power to investigate our hypotheses considering an estimated 50% dropout.

### Procedure

Participants approached the team via the ENYOY website or email address, university platforms, social media, advertisements, or a recruitment platform (Link2Trials) to show interest. As a response, contact details were collected, and the study information and consent forms were sent by post. Contact was made with the participant 5 days after the package was sent to conduct a short prescreening that was done digitally because of the COVID-19 pandemic (ie, via phone or Teams [Microsoft Corporation]; participants who were experiencing or receiving treatment for severe mental health issues were excluded and given information about other mental health care services) and to set up an intake appointment.

**Figure 1:**
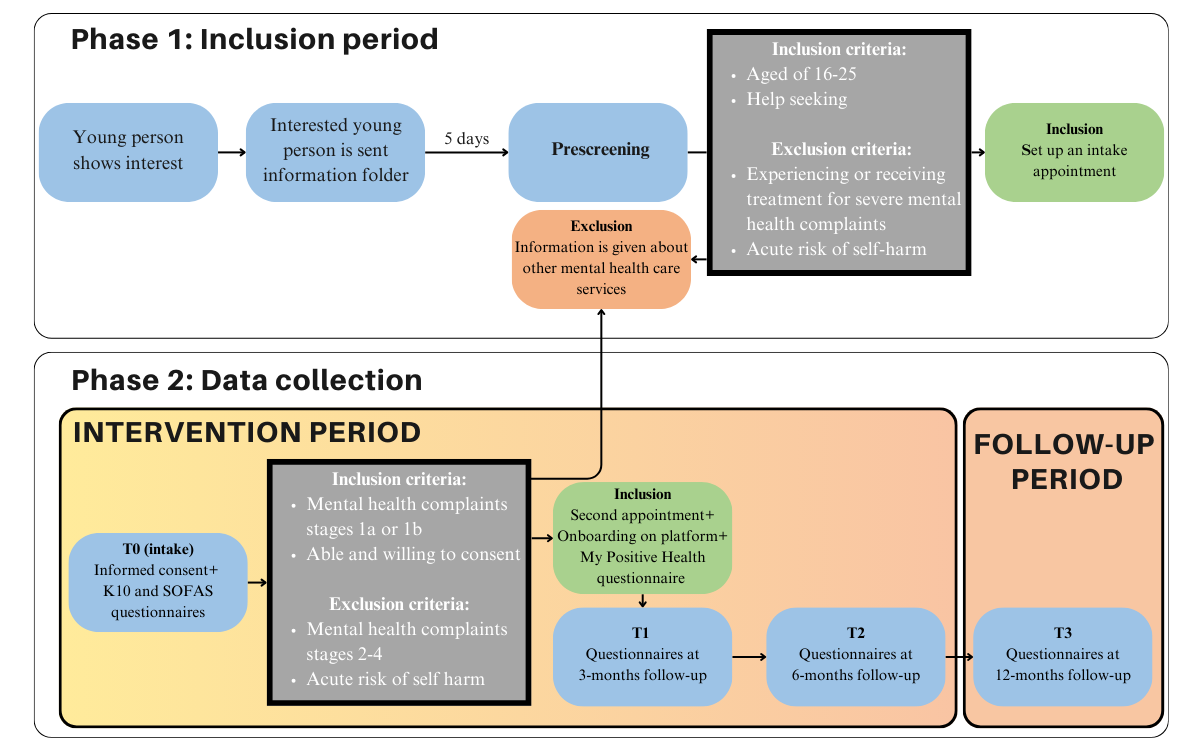
Participant timeline. K10: Kessler Psychological Distress Scale; SOFAS: Social and Occupational Functioning Assessment Scale.

During a 1-hour baseline assessment interview (intake), participants provided informed consent and were screened for eligibility using a semistructured interview and questionnaires (see the *Questionnaires* section) and by means of the operationalization of the clinical model by Hickie et al [21] (see the *Participants* section). Participants were informed that participation was voluntary and that they could stop participation at any moment. If this happened, reason or reasons for dropout were noted. The intake information was reviewed in a weekly consensus meeting with a (clinical or mental health) psychologist and peer workers to determine the clinical stage [21]. Noneligible participants were contacted to offer further guidance for other mental health care services, and eligible participants were contacted to schedule the second appointment. During this appointment, participants were “onboarded” on the ENYOY-platform and filled out the My Positive Health (MPH) questionnaire (see the *Questionnaires* section [76]), and biweekly coaching appointments were made (Figure 1). Participants were allowed to use the platform as much as they liked over the course of 6 months. Follow-up measurements were taken at 3, 6, and 12 months. All data were stored using the Castor electronic data capture software [80].

### Materials

#### Intervention

The platform contained the modules (building blocks) outlined in Textbox 2.

**Modules of the platform.**

**Guided therapy journeys**

- Each participant completed a questionnaire (based on the positive psychology framework [81,82]) and an algorithm tailored to a treatment journey specific to an individual's strengths and needs. On the basis of the preferences of an individual, together with a therapist, the therapy journey could be tailored even further (eg, changing the order of exercises, and adding or deleting exercise types or content), thereby enabling participants to modify the intervention to their personal needs and shape their own therapy journey. The activities entailed the following:
  Reflective actions, which are behavioral or cognitive experiments used to improve the ability to notice thought processes in order to build insight and self-awareness of these processes;
  Regular actions, which are behavioral experiments in real-world contexts used to generalize adaptive coping strategies and behaviors by increasing self-efficacy and by challenging cognitions;
  Therapy comics, which are engaging, powerful, and accessible means to understand and negotiate mental ill health challenges;
  Talking points, which are topics of discussion that provide an opportunity to share effective coping strategies to encourage social problem- solving and peer modeling and learning.

**Personalized therapy toolkit**

- A library of therapy work and favorite strategies.

**Safe digital social network**

- A moderated digital support network of all the included young people, there to support each other if and when they need it on their recovery journey.

**Professional digital support**

- Digital support from peer workers and clinical moderators.

The ENYOY-platform follows safety protocols based on the MOST intervention [83] that have been approved by 3 ethics committees and have been successfully implemented in 4 pilot studies and 2 randomized controlled trials (RCTs) [84,85]. The platform has an automated alert system for identifying increased risk of suicide or self-harm, and therapists screen for clinical risk information twice daily. High-risk words activate the safety protocol, following which the clinical moderator conducts a telephone risk assessment and, where necessary, implements one or more of the following procedures: (1) informing the general practitioner, (2) informing the nominated emergency contact, and (3) liaising with suitable emergency services. The platform also includes safety features such as a reporting function for users and visible 24/7 emergency numbers. Information and communications technology safety is in compliance with European laws and safety regulations developed in cooperation with a privacy officer from Amsterdam University Medical Centers.

#### Questionnaires

Questionnaires were administered at baseline and at the 3-, 6-, and 12-month follow-ups to assess the platform intervention (3- and 6-month measurements) and evaluate whether the initial effects were sustained over time (12-month measurement). The overall missing data rate was 22.54%. The overall level of functioning was measured using the Social and Occupational Functioning Assessment Scale (SOFAS) [75], which has a validated translation to Dutch [86]. The SOFAS consists of 15 open-ended questions (eg, “How is your contact with your family and/or partner?”). A 1-item rating ranges from 1 to 100 (1=inability to function; 100=superior functioning). It has excellent interrater reliability [87] (intraclass correlation coefficient=0.83).

Psychological distress was measured using the validated Dutch version [88] of the Kessler Psychological Distress Scale (K10) [74] self-report questionnaire. The K10 consists of 10 questions (eg, “during the past month, how often did you feel restless?”) that are scored on a 5-point Likert scale (1=*always*; 5=*never*). Higher total scores indicate more severe psychological distress. Strong psychometric qualities have been found, that is, good internal consistency (Cronbach α=.91), strong interitem correlation (0.350-0.659) [89], and high reliability (Cronbach α=.94) [88].

The Dutch self-report questionnaire MPH [76] assessed positive health. The MPH subscales measure daily functioning, physical functioning, mental well-being, participation, quality of life, and meaningfulness. For the purpose of this study, the mental well-being (eg, “I feel happy”), quality of life (eg, “I enjoy my life”), and meaningfulness (eg, “I know what things I would like to do in my life”) subscales were used. A total of 44 items were scored on a 10-point Likert scale (1=*not at all*; 10=*extremely*). The reliability was found to be good to very good (Cronbach α=.820-.933), and the discriminant validity was acceptable but with some overlap between domains [90]. An updated version of the instrument with improved validity is now available [76].

### Data Analysis

#### User Statistics

Data management and confidentiality measures have been described previously [24]. Age (into 2 groups based on age, with a median split at 21 y: 16-21 y and 22-25 y), educational level (lower, middle, and higher education [91]), and sex differences were assessed using chi-square analyses [49].

#### Mean Changes in Outcomes Over Time

A repeated measures ANOVA was used to assess changes over time (baseline and 3, 6, and 12 mo) for the K10 and SOFAS data. For the secondary analysis, the MPH data were analyzed using a repeated measures ANOVA to explore the changes over time. As the parameters of daily and physical functioning and participation are already included in the SOFAS measure [75], the mental well-being, quality of life, and meaningfulness subscales were used as outcome variables. All analyses were adjusted for age, sex, therapy activity, and community activity to determine whether the effects over time on the K10 and SOFAS scores were independent of educational level, sex, and participant involvement as common confounders. Post hoc pairwise comparisons with Bonferroni correction were performed to investigate the effects between each temporal condition. Post hoc analyses indicated sufficient statistical power (>0.9) [24]. Effect sizes were reported for the primary and secondary analyses (η_p_^2^) and post hoc pairwise comparisons (Cohen *d*). To investigate whether dropout and missing data (22.54%) influenced the results, we performed a sensitivity analysis using a repeated measures ANOVA on the complete data set (baseline; N=131). Multiple imputations (50 [92,93]) were used to fill in the missing values using both the dependent and time variables.

#### Reliable and Clinically Significant Change

To assess change indicators, reliable improvement (RCI) and belonging to a clinical versus nonclinical population (CSI) were computed individually on the sample [77] comparing the baseline versus 6-month measurements and the baseline versus 12-month measurements. For the RCI, a distinction was made between groups 1a and 1b to compare the course of mental health complaints. A moderate effect size of ≥0.5 was considered a significant change [77]. For the K10, the RCI was estimated at a 7-point change, and the CSI was estimated at 23 points (following the studies by Rickwood et al [49] and Slade et al [94]). For the SOFAS, the RCI was estimated at a 10-point change, and the CSI was estimated at 69 points [49]. Furthermore, potential variations in individual outcomes between clinical stages 1a and 1b were subject to exploratory investigation as the staging model indicates that these groups are at different risk stages [21,23].

#### Predictors for Improvement

Finally, we conducted a logistic regression analysis to investigate the potential protective effects (of independent variables [quality of life and meaningfulness]) at 6 months (after the intervention) in relation to progression (on dependent variables [psychological distress and psychosocial functioning]) at 12 months.

### Ethical Considerations

The study was reviewed and approved by the Medical Research Ethics Committee at Amsterdam University Medical Centers (NL66345.018.18). The participants provided written informed consent to take part in this study.

## Results

### Participants

**Figure 2:**
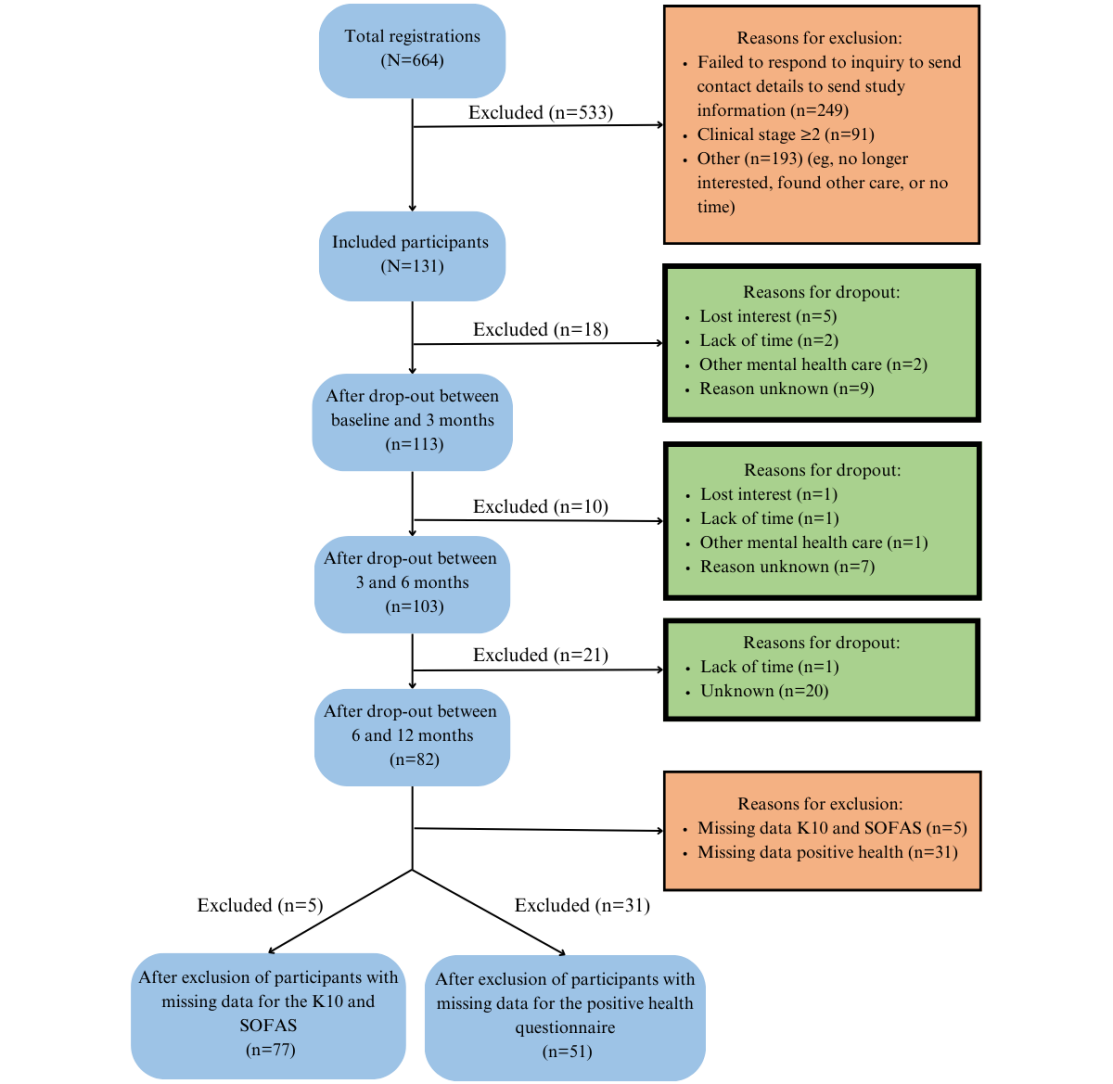
Distribution of participants (dropout flow). K10: Kessler Psychological Distress Scale; SOFAS: Social and Occupational Functioning Assessment Scale.

Of the interested young people, 37.5% (249/664) failed to respond or had personal reasons for not joining, and 42.8% (284/664) were excluded. A sample of 131 remained. Excluding participants with missing data resulted in a final sample of 77 for the primary measurements and 51 for the secondary measurements (Figure 2).

Of the participants, 88.5% (116/131) were female with an average age of 21.61 (SD 2.2) years, and 85.5% (112/131) had completed higher education. A total of 43.5% (57/131) were categorized as stage 1a and 56.5% (74/131) were categorized as stage 1b. Participants’ experiences of psychological distress ranged from “low” to “very high,” and their psychosocial functioning ranged from “serious impairment” to “good functioning.” Participants lived in different provinces in the Netherlands, with 89.3% (117/131) being Dutch or of Dutch and other ethnic and racial backgrounds (Table 1). Most found ENYOY via a recruitment platform (Link2Trials; 57/131, 43.5%), followed by social media (29/131, 22.1%), their general practitioner (6/131, 4.6%), family or friends (2/131, 1.5%), @ease center (1/131, 0.8%), or unknown (36/131, 27.5%). There were more female and relatively more highly educated participants (*P*<.001 in both cases). No differences in clinical stage 1a versus 1b were found (*P*=.16). A chi-square test indicated that there were no significant differences between dropouts and nondropouts on age (*P*=.62), educational level (*P*=.76), sex (*P*=.22), or clinical stage (*P*=.46). A 2-tailed independent-sample *t* test showed that there were no significant differences between dropouts and nondropouts in baseline K10 scores (*P*=.31), SOFAS scores (*P*=.96), or MPH scores (meaningfulness: *P*=.69; quality of life: *P*=.77; mental well-being: *P*=.34).

**Table 1:** Demographics of participants (N=131).

Variable		Values
Age (years), mean (SD)		21.63 (2.25)
**Clinical stage, n (%)**		
	1a	57 (43.5)
	1b	74 (56.5)
**Psychological distress^a^, n (%)**		
	Low^b^	4 (3.1)
	Moderate	31 (23.7)
	High	68 (51.9)
	Very high	28 (21.4)
**Psychosocial functioning^c^, n (%)**		
	Serious impairment^d^	3 (2.3)
	Moderate impairment	16 (12.2)
	Some impairment	66 (50.4)
	Slight impairment	43 (32.8)
	Good functioning	3 (2.3)
	Superior functioning	0 (0)
**Sex, n (%)**		
	Female	116 (88.5)
	Male	14 (10.7)
	Intersex	1 (0.8)
**Education^e^, n (%)**		
	Primary education	2 (1.5)
	Intermediate vocational education	17 (13)
	Higher vocational education	69 (52.7)
	University	43 (32.8)
**Ethnicity, n (%)**		
	Dutch or Dutch and other^f^	117 (89.3)
	Surinamese	6 (4.6)
	Other^g^	3 (2.3)
	Unknown	5 (3.8)

^a^Measured using the Kessler Psychological Distress Scale [74].

^b^Used cutoffs from the study by Slade et al [94].

^c^Measured using the Social and Occupational Functioning Assessment Scale [75].

^d^Used cutoffs from the study by Goldman et al [75].

^e^Highest level of education completed.

^f^Dutch and other ethnic and racial backgrounds.

^g^Ecuador and Colombia, Indonesia, and Iraq.

### User Statistics

At baseline, participants (N=131) were “onboarded” on treatment journeys with a main focus (young people can complete several different pathways; only the primary ones they started with are listed in this paper) on anxiety (84/131, 64.1%), social anxiety (34/131, 26%), or depressive complaints (10/131, 7.6%). A self-compassion pathway was developed and implemented after 1 year when clinical moderators noticed that young people struggled with negative self-image, with 2.3% (3/131) following this path. On average, youth visited the platform for 18.75 (SD 22.62; range 1-107) days, opened 42.29 (SD 46.78; range 0-278) therapy exercises, and were active on the community with 4.06 (SD 9.23; range 0-86) messages (posting and commenting on posts).

### Mean Changes in Outcomes Over Time

In the first analyses, we investigated whether platform use had an impact on psychological distress and daily functioning. This main analysis demonstrated a significant main effect of time on K10 scores (*P*<.001; η_p_^2^=0.239) and SOFAS scores (*P*<.001; η_p_^2^=0.318). These effects were not influenced by sex (*P*=.24 for psychological distress and *P*=.88 for psychosocial functioning), age (*P*=.76 and *P*=.48, respectively), community activity (*P*=.59 and *P*=.48, respectively), or therapy activity (*P*=.49 and *P*=.80, respectively).

**Figure 3:**
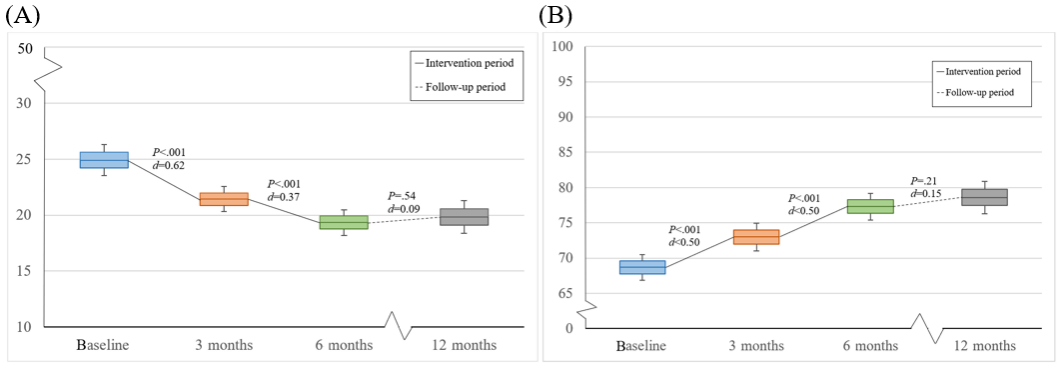
Changes over time for (A) psychological distress (Kessler Psychological Distress Scale [K10]) and (B) psychosocial functioning (Social and Occupational Functioning Assessment Scale [SOFAS]). Ranges for the scales’ total scores (minimum to maximum): 10 to 50 for the K10 and 0 to 100 for the SOFAS.

Subsequently, we analyzed and compared the effects of time at different intervals (baseline and 3, 6, and 12 mo) post hoc (Table 2 and Figure 3). Pairwise comparisons with Bonferroni correction showed significant improvements in K10 scores between baseline and 3 months (*P*<.001; *d*=0.62) and between 3 and 6 months (*P*<.001; *d*=0.37). No significant differences were found between 6 and 12 months (*P*=.54; *d*=0.09). Pairwise comparisons showed significant improvements in SOFAS scores between baseline and 3 months (*P*<.001; *d*=0.50) and between 3 and 6 months (*P*<.001; *d*=0.50). No significant differences were found between 6 and 12 months (*P*=.21; *d*=0.15).

**Table 2:** Mean, SD, mean difference, *P* value, and effect sizes (Cohen d) of the outcome measures psychosocial functioning (Social and Occupational Functioning Assessment Scale [SOFAS]), psychological distress (Kessler Psychological Distress Scale [K10]), and positive health parameters (quality of life, meaningfulness, and mental well-being subscales).

Outcome measure and time point			Values, mean (SD)		Time point comparison		Mean difference		*P* value		Cohen *d*
**SOFAS**											
	1^a^	68.68 (7.89)		N/A^b^		N/A		N/A		N/A	
	2^c^	72.97 (8.44)		2-1		4.30		<.001		0.50	
	3^d^	77.30 (8.23)		3-2		4.33		<.001		0.50	
	4^e^	78.58 (9.91)		4-3		1.29		.21		0.15	
**K10**											
	1	24.90 (6.18)		N/A		N/A		N/A		N/A	
	2	21.42 (4.97)		2-1		−3.48		<.001		0.62	
	3	19.32 (5.06)		3-2		−2.10		<.001		0.37	
	4	19.82 (6.35)		4-3		−0.49		.54		0.09	
**Quality of life**											
	1	5.83 (1.24)		N/A		N/A		N/A		N/A	
	2	6.26 (1.31)		2-1		0.43		.01		0.38	
	3	6.63 (1.32)		3-2		0.37		.02		0.28	
	4	6.81 (1.38)		4-3		0.18		.28		0.14	
**Meaningfulness**											
	1	6.39 (1.40)		N/A		N/A		N/A		N/A	
	2	6.80 (1.14)		2-1		0.41		.01		0.32	
	3	7.09 (1.39)		3-2		0.30		.02		0.23	
	4	7.31 (1.28)		4-3		0.21		.18		0.16	
**Mental well-being**											
	1	6.25 (1.16)		N/A		N/A		N/A		N/A	
	2	6.72 (1.10)		2-1		0.47		<.001		0.43	
	3	7.18 (1.04)		3-2		0.46		.002		0.42	
	4	7.17 (1.10)		4-3		0.01		.97		0.01	

^a^Baseline.

^b^N/A: not applicable.

^c^3-month measurement.

^d^6-month measurement.

^e^12-month follow-up.

To investigate whether dropout influenced our results, we performed additional sensitivity analyses on SOFAS and K10 data using linear mixed models (50 imputations), and the results were comparable for all outcome measures (*P*<.001 in all cases; effect size ranges for K10 scores: η_p_^2^=0.217-0.355; effect size ranges for SOFAS scores: η_p_^2^=0.110 and 0.211).

For the secondary analysis of MPH data, we examined whether the platform had an impact on positive health parameters. Repeated measures ANOVAs demonstrated a significant main effect of time on mental well-being (*P*<.001; η_p_^2^=0.140), quality of life (*P*=.03; η_p_^2^=0.062), and meaningfulness (*P*<.001; η_p_^2^=0.121). When adding age (*P*=.44 for mental well-being, *P*=.97 for quality of life, and *P*=.84 for meaningfulness), sex (*P*=.74, *P*=.78, and *P*=.56, respectively), therapy activity (*P*=.80, *P*=.36, and *P*=.46, respectively), and community activity (*P*=.58, *P*=.76, and *P*=.97, respectively) to the model, the effects remained essentially unchanged.

**Figure 4:**
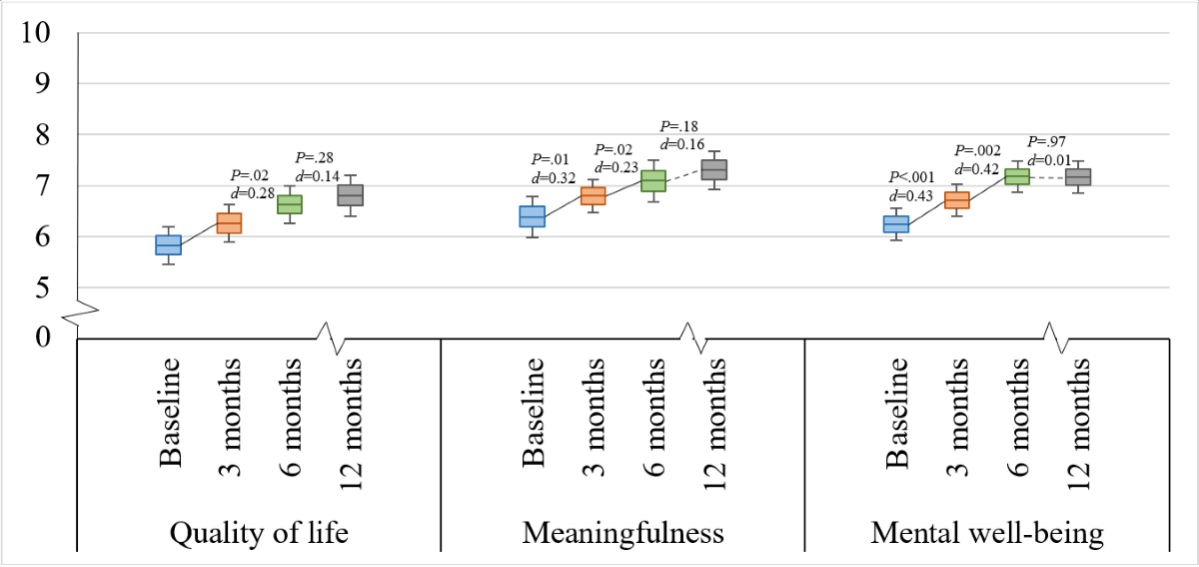
My Positive Health scores over time. Ranges for the subscales’ total scores (minimum to maximum): 0 to 10.

Subsequently, we conducted a post hoc pairwise comparison analysis to examine and compare the effects of time at different intervals (baseline and 3-, 6-, and 12-month measurements; Table 2 and Figure 4). The analysis showed significant improvements in mental well-being, quality of life, and meaningfulness between baseline and 3 months (*P*<.001 and *d*=0.43; *P*=.01 and *d*=0.38; and *P*=.01 and *d*=0.32, respectively) and between 3 and 6 months (*P*=.002 and *d*=0.42; *P*=.02 and *d*=0.28; and *P*=.02 and *d*=0.23, respectively). No differences were found in mental well-being, quality of life, or meaningfulness between 6 and 12 months (*P*=.97 and *d*=0.01; *P*=.28 and *d*=0.14; and *P*=.18 and *d*=0.16, respectively).

### Reliable and Clinically Significant Change

For the percentages of young people showing reliable and clinically significant changes, refer to Table 3.

**Table 3:** Proportion of young people showing reliable and clinically significant changes in psychological distress and psychosocial functioning between the baseline and 6-month measurements and between the baseline and 12-month measurements (N=131).

Measure, method, and clinical stage			Change category at 6 months (n=102), n (%)					Change category at 12 months (n=82), n (%)			
			Participants	Improvement	No change	Worsening	Participants		Improvement	No change	Worsening
**K10^a^**											
	**RCI^b^**										
		1a	50 (49)	19 (18.6)	28 (27.5)	3 (3)	36 (43.9)		19 (23.2)	17 (20.7)	0 (0)
		1b	52 (51)	20 (19.6)	29 (28.4)	3 (3)	46 (56.1)		18 (22)	20 (24.4)	8 (9.8)
	**CSI^c^**										
		1a and 1b	102 (100)	77 (75.5)	25 (24.5)	N/A^d^	82 (100)		63 (76.8)	19 (23.2)	N/A
**SOFAS^e^**											
	**RCI**										
		1a	48 (47.1)	26 (25.5)	19 (18.6)	3 (3)	36 (43.9)		22 (26.8)	12 (14.6)	2 (2.4)
		1b	54 (52.9)	25 (24.5)	25 (24.5)	4 (3.9)	46 (56.1)		17 (20.7)	26 (31.7)	3 (3.7)
	**CSI**										
		1a and 1b	102 (100)	91 (89.2)	11 (10.8)	N/A	82 (100)		75 (91.5)	7 (8.5)	N/A

^a^K10: Kessler Psychological Distress Scale.

^b^RCI: Reliable Change Index.

^c^CSI: Clinically Significant Index.

^d^N/A: not applicable; participants in the clinical population stay in the clinical category.

^e^SOFAS: Social and Occupational Functioning Assessment Scale.

Comparing the 6-month measurement to baseline with regard to psychological distress, participants in stage 1a reliably improved in 38% (19/50) of cases, did not change in 56% (28/50) of cases, and reliably worsened in 6% (3/50) of cases. Participants in stage 1b reliably improved in 38% (20/52) of cases, showed no change in 56% (29/52) of cases, and reliably worsened in 6% (3/52) of cases. Overall, 75.5% (77/102) of cases showed clinically significant changes, and 24.5% (25/102) of cases showed no change. Regarding psychosocial functioning, participants in stage 1a reliably improved in 54% (26/48) of cases, did not change in 40% (19/48) of cases, and reliably worsened in 6% (3/48) of cases. Participants in stage 1b improved in 46% (25/54) of cases, showed no change in 46% (25/54) of cases, and reliably worsened in 7% (4/54) of cases. Overall, 89.2% (91/102) of cases showed clinically significant changes, and 10.8% (11/102) of cases showed no change.

Comparing the 12-month measurement to baseline with regard to psychological distress, participants in stage 1a reliably improved in 53% (19/36) of cases and did not change in 47% (17/36) of cases, and no cases reliably worsened. Participants in stage 1b reliably improved in 39% (18/46) of cases, showed no change in 43% (20/46) of cases, and reliably worsened in 17% (8/46) of cases. Overall, 77% (63/82) of cases showed clinically significant changes, and 23% (19/82) of cases showed no change. Regarding psychosocial functioning, participants in stage 1a reliably improved in 61% (22/36) of cases, did not change in 33% (12/36) of cases, and reliably worsened in 6% (2/36) of cases. Participants in stage 1b improved in 37% (17/46) of cases, showed no change in 57% (26/46) of cases, and reliably worsened in 7% (3/46) of cases. Overall, 91% (75/82) of cases showed clinically significant changes, and 9% (7/82) of cases showed no change.

### Predictors for Improvement

Improvement in both psychological distress and social functioning was predicted at the 6-month measurement by higher psychological distress (*P*<.001) and lower social functioning (*P*=.04) at baseline, respectively. This means that both severity of mental health complaints and impairment of social functioning predicted greater improvement in young people. In addition, age (*P*=.24), sex (*P*=.89), educational level (*P*=.19), and clinical stage (*P*>.99) did not predict improvement.

In an additional set of exploratory analyses, we investigated the potential protective effects in relation to the progression of symptoms between 6 and 12 months. Logistic regression analyses showed that higher levels of meaningfulness (odds ratio 3.59, 95% CI 1.22-10.53) but not quality of life at 6 months (after the intervention) were associated with a lower risk of complaints worsening at the 12-month follow-up.

## Discussion

### Principal Findings

The aim of this study was to investigate the outcomes of the ENYOY-platform in relation to the following parameters: psychological distress, psychosocial functioning, and positive health parameters (such as well-being and quality of life [24]). Our hypothesis was that ENYOY would attain similar results of improvement in psychological distress and psychosocial functioning as have been found for the young people visiting the Headspace centers in Australia [49], as well as a positive change in positive health parameters. It was expected that spending more time on the platform and being more active in the digital community would be associated with increased improvements.

The main analysis revealed significant and substantial improvements in psychological distress and psychosocial functioning over time (large effect sizes) independent of sex, age, therapy activity, or community activity (negligible to small effect sizes). This contradicts the initial hypothesis that spending more time on the platform or being more active in the digital community would lead to greater improvements. Regardless of use frequency, the platform consistently had large effects among young people. In addition, the secondary analysis showed significant improvements in well-being, quality of life, and meaningfulness (large, medium, and large effect sizes, respectively) independent of sex, age, therapy activity, or community activity (negligible to small effect sizes). Notably, significant improvements in all outcome measures occurred within the first 3 months (moderate effect sizes for psychological distress and psychosocial functioning, small to moderate effect sizes for mental well-being and quality of life, and small effect sizes for meaningfulness), with further significant increases in the subsequent 3 months (moderate effect sizes for psychosocial functioning, small to moderate effect sizes for psychological distress and mental well-being, and small effect sizes for quality of life and meaningfulness), and remained stable after active platform use (ie, 12-month follow-up; very small to negligible effect sizes). These findings highlight the platform’s effects in decreasing psychological distress, improving psychosocial functioning, and improving positive health parameters independent of individual characteristics or engagement levels. Moreover, there were no age effects, which implies that a broad age range (ages of 16-25 y) could benefit from this intervention. The sustained positive outcomes over time underscore the potential long-term benefits of using this intervention. This is also in line with a previous qualitative study that showed that young people perceived the ENYOY-platform to be a user-friendly, safe, accessible, and inclusive initiative that helped alleviate their mental health complaints and enhanced their overall quality of life [67].

Interestingly, in line with previous research [68-70], we initially anticipated similar medium effect sizes. However, the observed effect sizes in our study turned out to be even higher. A possible explanation for this difference is the longer engagement period of Dutch young people on the platform compared with the Australian studies (6 vs 3 mo). The extended duration likely contributed to the enhanced effects, as evidenced by the significant improvements over additional months. Another factor could be the homogeneity of our participant group, with a majority of female and higher-educated individuals. In contrast, user motivation—measured through platform visits—was not notably higher than that in previous studies [66], and the amount of community and therapy activity exhibited a wide variation among participants. Discrepancies with Australian studies [49,66] could also stem from differences in concern profiles or severity. The Australian studies did not select participants based on concern severity, possibly including individuals with a higher severity of complaints (clinical stage ≥2). Furthermore, the methodology of the study by Rickwood et al [49], which analyzed K10 and SOFAS scores at specific time points, makes direct comparisons challenging as it involved physical center visits rather than a web-based platform.

Of interest was the unexpected finding that the effects of the platform were independent of platform use. This could potentially be attributed to the platform’s unique composition, consisting of 4 main modules: therapy exercises, conversations with peer workers, sessions with psychologists, and a community for peer-to-peer support. Guided by the principles of self-determination theory [95,96], we empowered young individuals to determine their own use patterns for each module, tailoring their engagement based on their specific needs. This individualized approach enabled young people to create their own optimal use of the platform’s modules, rendering the measurement of therapy or community activity alone redundant for capturing the platform’s overall impact.

### Individual Change

In line with the main analysis, clinical and reliable changes were observed in a large portion of the sample at the 6-month follow-up. Regarding psychological distress, 38% (19/50) of young people in stage 1a and 39% (18/46) of young people in stage 1b reliably improved. Similarly, for psychosocial functioning, 54% (26/48) of young people in stage 1a and 46% (25/54) of young people in stage 1b showed reliable improvement. The percentage of individuals who experienced a worsening of complaints (3/50, 6%) and functioning (4/54, 7%) was minimal. A substantial majority of cases (77/102, 75.5% for psychological distress and 91/102, 89.2% for psychosocial functioning) no longer met the criteria for clinical levels of complaints. An interesting finding was that approximately one-sixth of individuals in clinical stage 1b (8/46, 17%) experienced increased psychological distress at the follow-up, whereas no one in stage 1a deteriorated. This aligns with previous research indicating a clear distinction between the 2 stages [25-27]. Individuals in the latter stage may require continued care. In Australia, young people are initially provided with an “active” intervention period of 3 months during which clinicians reach out to them weekly. After this phase, the intervention shifts to a more passive approach, allowing young people to use the platform and seek help as needed. This approach shows promise for the 1b group, which is at a higher risk of deterioration, by ensuring ongoing support while promoting independence. Despite increased psychological distress, individuals in stage 1b maintained their social and occupational functioning, which may indicate that they are more accepting of their mental health symptoms.

### Explorative Analysis

It is revealed that a greater severity of mental health complaints and impairment of social functioning predicted greater reliable change in young individuals. This finding may be attributed to the presence of ceiling effects for social functioning and floor effects for mental health complaints, which are more likely to occur among young people with milder mental health complaints [97,98]. In other words, young individuals with higher initial levels of mental health complaints have more room for improvement, leading to greater positive changes over time. This finding challenges the previous assertion that the lower effect size of studies in Australia could be due to the inclusion of young people with higher-severity complaints. Although we can only speculate, this might suggest that the platform may also have positive effects on young people with more severe mental health complaints (beyond clinical stage 1b). Explorative analyses further revealed that, for individuals in stage 1b, higher levels of meaningfulness at 6 months (after the intervention) predicted a lower change in worsening at 12 months (follow-up), which could indicate that meaningfulness is a protective factor. For future interventions, this could be taken into account, for example, by providing the subgroup in stage 1b with low levels of meaningfulness through more care or continuity of care or offering additional interventions aimed at increasing meaningfulness.

Despite exploring numerous variables, no specific indications were found regarding any particular variable that may have contributed to an increased likelihood of dropout. Previous studies have identified several predictors of dropout, such as psychosocial difficulties, symptom severity [99], being young, being male, the involvement of more than one therapist in treatment, having no history of psychiatric disorders [100], lower social support [101], and lower change motivation [102]. A comprehensive review of multiple studies on dropout characteristics concluded that predictors may vary across studies and samples and over time [103], making it challenging to draw conclusions in general.

### Strengths and Limitations

A notable limitation of this study is the identified sample bias, which favored White female individuals with higher education. Young people with a different ethnic background represented only 6.9% (9/131) compared with the 30% among young people in the Netherlands [104], an explanation being the platform’s sole availability in Dutch, thereby excluding individuals who speak other languages. Similarly, lower education was underrepresented compared with the equal distribution in the Netherlands [105]. Although different recruitment measures were implemented to obtain a diverse sample [24], certain young people were not reached. It is worth noting that similar studies have also found a bias toward female individuals with higher education [64]. Consequently, caution should be exercised when interpreting the results as they may only reflect an effect among this specific group of young people. No significant interaction effects of sex were observed, suggesting that the effects may extend to male individuals as well. However, to draw conclusive statements about male participants, a larger proportion of male individuals in the sample would be required. To comprehensively assess the intervention’s effects, future research should incorporate more diverse samples [106]. In addition, questions might arise about the platform’s suitability for a wider age range than 16 to 25 years. Notably, young people significantly influenced the ENYOY design across all phases, ensuring its relevance to the target population. Although our initial intention was to encompass young individuals aged 12 to 25 years [24], a decision was made to pilot the platform with the age range of 16 to 25 years based on recommendations from the institutional review board. Furthermore, the absence of a statistically significant age effect on the outcome measures suggests that age did not significantly influence the results. Therefore, it is expected that the platform accommodated a wide range of developmental ages, including 16 to 25 years, to a significant extent. It is important to note that accommodating participants aged <16 years in future studies would require platform adjustment [24,106].

Finally, it is important to acknowledge that this study was not an RCT, resulting in a lower level of evidence [107]. The extent to which the observed effects are attributable to the intervention or other factors remains unclear. It is essential to consider that these effects may be a result of other interventions. We adopted a participatory within-group design for ongoing platform enhancement based on user feedback, recognizing that starting with an RCT in implementation research could oversimplify real-world patient diversity, become time-consuming, and have strong internal but limited external validity [108]. Rapid technological advancements in web-based interventions could also risk making RCT results obsolete upon publication [109]. Although we acknowledge the importance of a more robust RCT design in the future, including a larger number of participants and a control group after the broader implementation of the ENYOY, we opted for flexibility to adapt quickly to user feedback and capture the complexities of real-world use. This choice involved a trade-off, potentially affecting the sample’s representativeness, but allowed us to balance scientific rigor with accommodating the diverse and evolving needs of our user base [109]. Nonetheless, the observed improvements during platform use and their sustained stability after 6 months, combined with the consistent effectiveness found in >13 years of research (including RCTs) in Australia [65,66,68-72], strongly suggest the potential benefits of using the platform. This study has established a foundation, and the subsequent step is to validate these findings through an RCT to ensure a higher level of evidence.

A strength of this study was its high retention rate and statistical power. Over the course of a year, the study had a dropout rate of only 62.6% (82/131), and the missing data accounted for only 22.54%. These figures surpass those found in most comparable research studies, which typically report dropout rates of up to 73% [64]. Group-level analysis enabled the examination of overall trends and patterns, whereas individual-level analysis provided valuable insights into each participant’s response [110]. This comprehensive approach could enhance decision-making and facilitate the future customization of the intervention to address the diverse needs of young people. Finally, the repeated measures design offered valuable insights into the trajectory of improvements over time and the sustainability of the effects following the intervention.

### Recommendations

This trial has demonstrated that the ENYOY-platform holds promise as an intervention for addressing emerging mental health complaints among young people in the Netherlands. By addressing these complaints, the platform has the potential to reduce the burden of mental illness and associated costs among youth [111-113], potentially alleviating long waiting lists for mental health care, and offers a solution to bridge the gap between child, adolescent, and adult psychiatry [15,114,115]. To reach a wider audience, the platform could expand its language options and incorporate features for individuals with low literacy levels. For future projects, the inclusion of external informants (such as family and friends) should be considered to explore the potential benefits of involving them in the system, which could contribute to treatment customization, offer a holistic view of individual complaints, provide context, and possibly enhance therapy outcomes [106]. This trial represents a significant milestone in the implementation of a transdiagnostic, digital, and clinically and peer-moderated indicative prevention treatment platform for youth with emerging mental health complaints in the Netherlands [24]. Furthermore, the success of this trial opens up possibilities for the platform’s implementation in other countries as well.

### Future Research

This study has laid the groundwork for enhanced clinical research in the Netherlands using the ENYOY-platform. The results provide support for progressing to an RCT. In addition, it would be relevant to expand access to the platform to other populations. For example, in Australia, young people with mild to more severe problems have been granted access to the platform via youth mental health services to enhance face-to-face care during waiting periods, supplement face-to-face treatment, and support follow-up care (eg, relapse prevention) [66,71]. To inform decision-making regarding the criteria for offering ongoing care, it is important to calculate cutoff scores for meaningfulness for stage 1b individuals. This could help identify those who would benefit from ongoing support, thus enabling targeted interventions. In addition, future research could further investigate which factors predict dropout to develop more targeted interventions and support systems that effectively address the underlying causes and mitigate the risk of dropout. A recent study in Australia demonstrated the cost-effectiveness of the platform, which could be repeated for the Dutch situation [116]. Finally, as the most vulnerable period for developing mental health complaints is between the ages of 12 and 25 years [117,118], there is a need to include individuals aged 12 to 15 years in the trials (on the same platform or a customization of the current platform that better matches this age group).

## Abbreviations

CBT: cognitive behavioral therapy
CSI: Clinically Significant Index
ENYOY: Engage Young People Early
K10: Kessler Psychological Distress Scale
MOST: Moderated Online Social Therapy
MPH: My Positive Health
RCI: Reliable Change Index
RCT: randomized controlled trial
SOFAS: Social and Occupational Functioning Assessment Scale

## Data Availability

The data sets generated during and/or analyzed during this study are available from the corresponding author on reasonable request.
